# *Lepidium Sativum *Extract Alleviates Reproductive and Developmental Toxicity in Polycystic Ovary Syndrome Induced by Letrozole and High-Fat Diet in Rats

**DOI:** 10.1007/s43032-025-01820-y

**Published:** 2025-03-06

**Authors:** Mariam Ahmed Moustafa, Ayman Saber Mohamed, Ahmed Imam Dakrory, Mennatallah H. Abdelaziz

**Affiliations:** Zoology Department, Faculty of Science, Cairo University, Giza, Egypt

**Keywords:** Polycystic ovary syndrome, Reproductive toxicity, Developmental toxicity, Letrozole, *Lepidium sativum*, Oxidative stress

## Abstract

Polycystic ovary syndrome (PCOS) is a common endocrine disorder characterized by irregular menstrual cycles, hyperandrogenism, and polycystic ovarian morphology, leading to infertility and an increased risk of metabolic dysfunction. This study investigated the protective effects of *Lepidium sativum* seed extract (LSSE) on fertility and pregnancy outcomes in a rat model of PCOS induced by letrozole and a high-fat diet (HFD) (68% regular rat food pellets, 6% maize oil, 20% milk powder, and 6% ghee). PCOS was induced by oral administration of letrozole, an aromatase inhibitor, (1 mg/kg in 2% DMSO) along with an HFD for four weeks and the results were compared to the metformin antidiabetic agent. Sixty rats were divided into five groups (*n* = 12): control, PCOS, PCOS + LSSE (250 mg/kg), PCOS + LSSE (500 mg/kg), and metformin (200 mg/kg). Half of the rats were euthanized after four weeks, while the remaining rats were housed with males (1 male: 2 females) for testing pregnancy outcomes. LSSE led to remarkable improvements in body weight, glucose, sex hormonal balance, liver and kidney functions, antioxidant and anti-inflammatory systems, mating and fertility indices, pregnancy outcomes, and fetal morphology. Histologically, LSSE treatment reduced ovarian cyst number and size, and enhanced the blood vessel, Graafian follicle, and endometrial morphologies. LSSE showed dose-dependent effects, with the high-dose demonstrating superior results in the biochemical parameters, while the low-dose showed the most promising pregnancy outcomes. LSSE exhibits potent protective effects against PCOS, surpassing metformin in several aspects. LSSE acts through multi-mechanistic action, including anti-inflammatory, antioxidant, metabolic, and hormone-balancing properties, along with its positive impact on fertility, suggesting that LSSE could be a promising natural alternative for PCOS management.

**Graphical Abstract:**

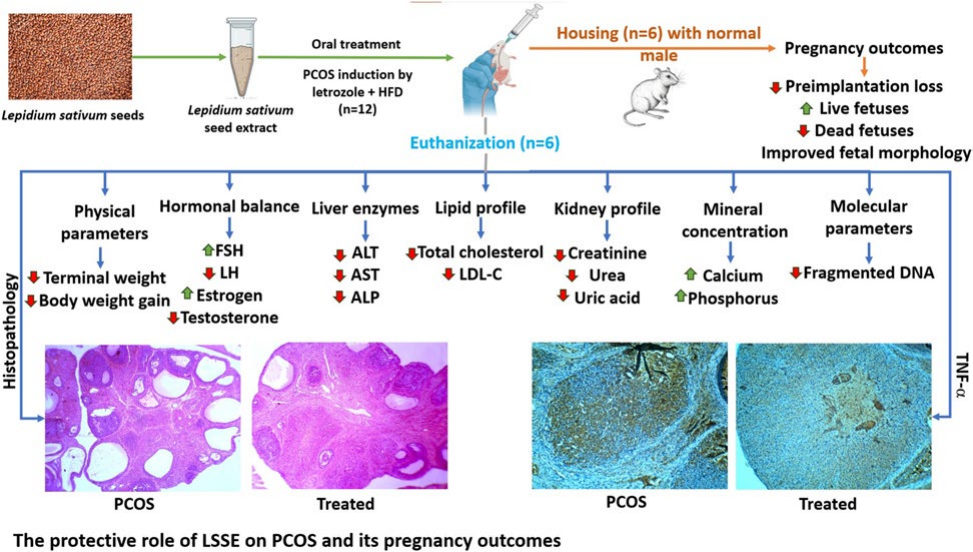

## Introduction

Polycystic ovarian syndrome (PCOS) is a prevalent endocrine disorder affecting 5–15% of reproductive-age women and represents a major cause of female infertility [1]. This syndrome is characterized by irregular menstruation, hormonal imbalance, including hyperandrogenism, and altered polycystic ovarian morphology, as evidenced by ultrasound imaging [2]. PCOS patients often experience metabolic dysfunction as insulin resistance and face increased risks of type 2 diabetes, gynecological malignancies, and increased risk of pregnancy complications [3].

The pathophysiology of PCOS involves a complex interplay of hormonal dysregulation, chronic inflammation, insulin resistance, and hyperandrogenism, influenced by genetic predisposition, environmental factors, and dietary habits [4]. The hormonal profile in PCOS patients typically shows elevated testosterone, insulin, luteinizing hormone (LH), and estrogen, with a concurrent decrease in follicle-stimulating hormone (FSH) and progesterone [5].

Insulin resistance, present in a significant proportion of PCOS patients, plays a crucial role in the syndrome's progression [6]. It leads to hyperinsulinemia, intensifying androgen production by theca cells through up-regulation of LH-binding sites [7]. Additionally, insulin decreases sex hormone binding globulin (SHBG), contributing to hyperandrogenism [8]. That creates a self-reinforcing loop between hyperinsulinemia and hyperandrogenemia, as excess androgens stimulate visceral adipose tissue to generate free fatty acids, exacerbating insulin resistance [9].

The animal models efficiently elucidated the pathogenesis of PCOS and tested potential treatments [10]. A well-established approach involves the use of letrozole, an aromatase inhibitor, in combination with a high-fat diet to induce PCOS-like features in rats [11]. This model replicates vital aspects of the syndrome, including anovulation, hormonal imbalances, metabolic disturbances, and insulin resistance [12].

The existing pharmacological approaches for PCOS, although advantageous, may suffer from inadequate efficacy or undesirable side effects [13]. Given these constraints, there is an increasing interest in investigating natural substances for treating PCOS. *Lepidium sativum* (garden cress), an annual herb with a substantial nutritional profile, has attracted interest for its possible therapeutic benefits [14]. Its seeds are rich in bioactive compounds such as proteins, fatty acids, and phenolic compounds, which have been traditionally associated with reproductive health and metabolic regulation, which may influence the critical pathways implicated in PCOS pathophysiology [15]. The *Lepidium sativum* seed extract (LSSE) exhibits diverse biological properties, including anti-inflammatory, antioxidant, and antidiabetic activities, these properties are particularly relevant to PCOS, which is characterized by inflammation and metabolic dysfunction [16]. Despite this promising profile, LSSE's effects on PCOS remain understudied. Therefore, the current study aims to evaluate the protective effect of LSSE on female infertility caused by PCOS and pregnancy outcomes in female adult rats.

## Materials and Methods

### Chemicals and Reagents

Letrozole (Techno Pharma, Egypt), metformin (Minapharm, Egypt), serum hormone ELISA kits (Autobio Diagnostics, China), biochemical assessment and oxidative stress kits (Biodiagnostic Company, Egypt), and tumor necrosis factor-α histology assay kit (Creative Biolab Company, USA), were used.

### Preparation of LSSE

*Lepidium sativum* seeds were obtained from the local herb market (Cairo, Egypt). The aqueous LSSE was prepared according to [17].

### Characterization of LSSE

#### High-Performance Liquid Chromatography (HPLC)

Agilent 1260 series HPLC analyzers were utilized. The Zorbax Eclipse Plus C8 column was used to perform the separation. At 0.9 ml/min flow rate, water (A) and 0.05% trifluoroacetic acid in acetonitrile (B) were the mobile phase. The linear gradient mobile phase was designed as follows: 0 min (82% A), 0–1 min (82% A), 1–11 min (75% A), 11–18 min (60% A), 18–22 min (82% A), 22–24 min. Multi-wavelength detector monitoring was done at 280 nm. A 5 μl injection volume was used for each sample solution. The column maintained constant temperature at 40 °C.

#### Determination of the Anti-Inflammatory Activity (Human Red Blood Cell (HRBC) Membrane Stabilization Assay)

The standard method used for the in vitro anti-inflammatory evaluation of the extract was described by Yesmin et al. [18].

Calculation

$$\%Inhibtion\;of\;hemoloysis=100\;\times\;\left(1-\frac{{OD}_2-{OD}_1}{{OD}_3-{OD}_1}\right)$$

where, ${OD}_{1}$ is the test sample unheated; ${OD}_{2}$ is the test sample heated and ${OD}_{3}$ is the control sample heated.

#### Determination of the Antioxidant Activity (2, 2-Diphenyl-1-Picrylhydrazyl (DPPH) assay)

This assay was conducted as described by Chatoui et al. [19].

### Experimental Animals

A total of 60 female and 15 male Wistar albino rats weighed approximately (180–200 g) were used in the present study. The rats used in the study were acquired from the National Research Center (Giza, Egypt). The rats were housed in a temperature and humidity-controlled environment and given food and water ad libitum.

### Experimental Design

After one week of acclimatization, 60 female rats were randomly divided into five groups (*n* = 12), and their initial body weight was recorded.

- **Group 1 (control):** rats were given 2% DMSO, distilled water orally (2 h between 2 doses), and a regular diet for four weeks.
- **Group 2 (PCOS):** rats were given letrozole (1 mg/kg in 2% DMSO) and distilled water orally along with an HFD for four weeks [20].
- **Group 3 (PCOS + LSSE low dose):** rats were given letrozole (1 mg/kg in 2% DMSO) and LSSE (250 mg/kg) [21] orally, along with a HFD for four weeks.
- **Group 4 (PCOS + LSSE high dose):** rats were given letrozole (1 mg/kg in 2% DMSO) and LSSE (500 mg/kg) [21] orally, along with a HFD for four weeks.
- **Group 5 (PCOS + Metformin):** rats were given letrozole (1 mg/kg in 2% DMSO) and metformin (200 mg/kg) [22] orally, along with a HFD for four weeks.

After four weeks, six rats from each group were weighed for their terminal weights and then euthanized on day 30 using the exsanguination procedure under anesthesia. The remaining female rats in each group were mated with healthy males. The detection of sperm in vaginal smears established the zero-day of fertilization, and the female was classified as pregnant, with the day of sperm detection designated as the initial day of gestation (GD1) [23]. On day 20 of gestation, they were euthanized using the same method mentioned earlier and sectioned by cesarean incision.

### Physical Parameters

Rats' weight was measured weekly for follow-up. The average weight gain of the rat's body weight was calculated. After euthanasia, the ovary was weighed, and the difference in ovary size between groups was observed.

### Sample Preparation

The blood samples collected by exsanguination were centrifuged at 3000 rpm for 20 min. The collected serum was stored at −20°C until used for biochemical assays. Regarding the histological examination, ovarian and uterine tissues were carefully dissected and stored in a 10% formalin solution. For the oxidative stress analysis, the ovarian and uterine tissues were homogenized (10% w/v) in ice-cold 50 mM phosphate buffer (pH 7.4). The homogenate was centrifuged at 3000 rpm for 15 min at 4°C, and the resultant supernatant was used. The ovarian and uterine tissues were homogenized in lysis buffer (pH 8) for the DNA fragmentation assay.

### Serum Biochemical Analyses

The serum concentration of luteinizing hormone (LH), follicle-stimulating hormone (FSH), estrogen, and testosterone hormones were assayed via the ELISA technique according to the ELISA kits. The serum glucose, aspartate aminotransferase (AST) and alanine aminotransferase (ALT), alkaline phosphatase (ALP), total protein (TP), albumin, total cholesterol (TC), low-density lipoprotein cholesterol (LDL-TC), high-density lipoprotein cholesterol (HDL-TC), urea, uric acid, lactate dehydrogenase (LDH), calcium, and phosphorus were determined according to the manufacturer's instructions.

### Determination of Oxidative Stress Biomarkers

Malondialdehyde (MDA), glutathione reduced (GSH), nitric oxide (NO), and catalase (CAT) were determined according to the manufacturer's instructions (Giza, Egypt).

### Determination of DNA Fragmentation Using PEG/ Hoechst Fragmentation Assay

DNA fragmentation was evaluated based on the fluorometric method of Ioannou and Chen [24].

### Histopathological Examination

The fixed ovarian and uterine tissues were washed, dehydrated, and embedded in paraffin wax. The tissues were sectioned 5–7 μm thick and stained with hematoxylin and eosin (H&E) for histopathological examination. A light microscope was used to examine the slides and photomicrographs of the ovarian and uterine tissues were taken at × 40 and × 100 magnifications. The number of ovarian follicles/sections, including antral, Graafian, corpora lutea, atretic, and cystic follicles in the ovary and endometrial glands/section in the uterus were counted. Also, the diameter of cystic follicles in the ovary and the average uterine wall thickness were measured using Image J software.

### Evaluation of Tumor Necrosis Factor-alpha (TNF- α)

For immunostaining, ovarian and uterine tissues were cut into positive slides, deparaffinized, rehydrated, and exposed to heat-induced antigen retrieval step for 15 min, followed by blocking steps for protein and endogenous peroxidases using BSA and hydrogen peroxide, respectively. After washing in PBS, tissue slides were incubated with primary antibody rabbit polyclonal anti-mouse phospho-RELA (S536) antibody Anti-TNF (rabbit polyclonal IgG, 100 μg/ml, 1:50 dilution) for 12 h at the refrigerator. HRP-labeled secondary antibody was applied for 2 h at room temperature. After washing, the DAB-substrate kit was utilized to produce the color. Control slides were formed by deletion of the primary antibody. Positive expression was assessed as area % using image j software.

### Female Fertility Assessment

After 30 days of treatment, we cohabited the female rats with healthy adult male rats (2:1). The maximal duration of pairing was one week to assess the mating index, which is used to indicate the incidence of copulation through observing sperm-positive females, while the female fertility index implies successful impregnation. Mating and fertility indices were estimated [25].

### Assessment of Maternal Toxicity

Females with successful copulation were isolated, observed daily to determine any behavioral changes, and weighed weekly. The rats were weighed on the first and 20th days of gestation.

### Analysis of Pregnancy Outcomes

The pregnant rats were euthanized on day 20 of gestation, and the offspring were delivered via cesarean section. The corpora lutea in the right and left ovaries were counted in all the study groups. The uterus was taken out, weighed, and the implantation sites were counted and photographed. The documentation included the count of live and dead fetuses and their locations. Every placenta was weighed. The preimplantation and postimplantation loss indices were calculated according to Burdan *et al*. (2005). Each fetus was weighed and examined for any external malformations.

### Statistical Analysis

The statistical package for social science (SPSS 25) was used for statistical analysis. For multiple comparisons, one-way analysis of variance (ANOVA) was employed, while independent samples Kruskal–Wallis were applied to the scores and percentages. The Duncan post hoc test followed both. The means ± standard errors of means were used to present the data. The differences between the groups were considered statistically significant when *p* < 0.05. Graphs were drawn using the software GraphPad Prism version 8.

## Results

### Characterization of Lepidium Sativum Seed Extract (LSSE)

#### High-Performance Liquid Chromatography (HPLC)

**Fig. 1:**
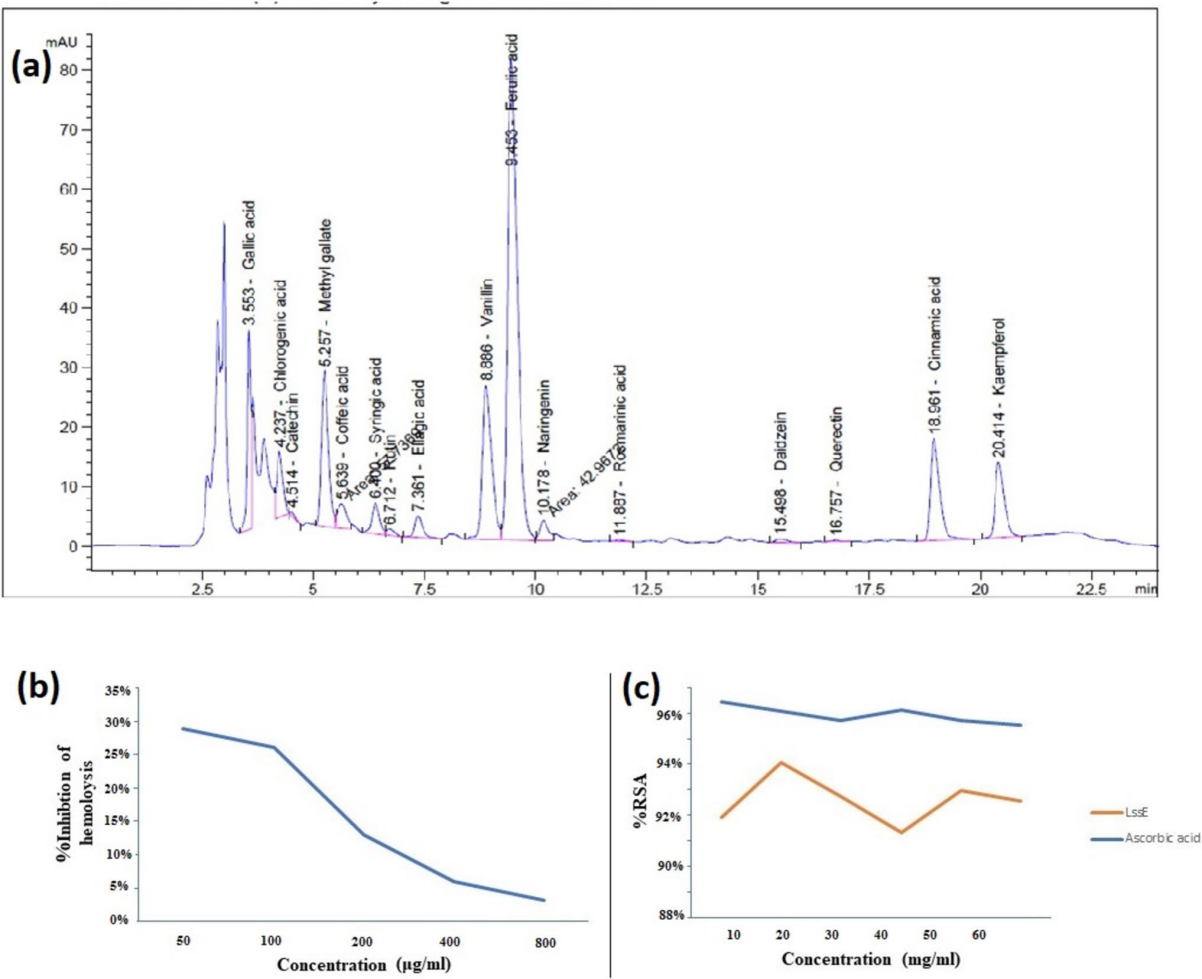
**a** High-performance liquid chromatography-photodiode (HPLC) chromatogram of bioactive molecules in LSSE, **b** anti-inflammatory activity of LSSE by heat-induced hemolysis method and (**c**) antioxidant activity of LSSE by DPPH method

The analysis of the component of the LSSE was carried out by HPLC, as presented in Table 1. The analysis indicated that LSSE contained several phenolic and flavonoids compounds, such as ferulic acid, methyl gallate, vanillin, chlorogenic acid, gallic acid, cinnamic acid, ellagic acid, and kaempferol (Fig. 1a).

**Table 1:** High-performance liquid chromatography-photodiode (HPLC) chromatogram of bioactive molecules in LSSE

Molecule	RetTime (min)	Area	Amount (µg/g)
Gallic acid	3.55	173.88	636.56
Chlorogenic acid	4.24	104.68	729.35
Catechin	4.51	4.65	50.00
Methyl gallate	5.27	284.38	795.58
Coffeic acid	5.64	57.74	148.11
Syringic acid	6.40	63.08	185.51
Rutin	6.71	13.79	103.23
Ellagic acid	7.36	44.72	227.16
Vanillin	8.89	386.30	700.54
Ferulic acid	9.45	1230.59	3571.78
Naringenin	10.18	42.97	198.28
Rosmarinic acid	11.89	3.58	17.38
Querectin	16.76	3.95	24.61
Cinnamic acid	18.96	246.87	239.24
Kaempferol	20.41	177.45	223.05

#### Determination of the Anti-Inflammatory Activity

All concentrations (50–800 μg/ml) of LSSE showed moderate to weak anti-inflammatory properties regarding RBC heat-induced hemolysis. The percentage of hemolysis inhibition ranged from 3 to 29%. Lower concentrations exhibited more potent anti-inflammatory activity than the higher ones (Fig. 1b).

#### Determination of the Antioxidant Activity

The antioxidant activity was assessed at varying concentrations (10–60 mg/ml). All concentrations of LSSE showed intense free radical scavenging activity, as evident from the DPPH assay. A 20 mg/mL concentration of LSSE displayed the highest antioxidant activity at 94.4% (Fig. 1c).

### Physical Parameters

The PCOS rats significantly increased (*p* < 0.05) in terminal weight, body weight gain, and the average weight of ovaries compared to the control rats. However, the treatment with LSSE or metformin showed a significant decline in the terminal and body weight gain in a dose-dependent manner. In contrast, no significant change was recorded regarding the average ovary weight compared to the PCOS rats (Table 2).

**Table 2:** Physical parameters of rats in different groups examined

	Control	PCOS	LSSE (250 mg/kg)	LSSE (500 mg/kg)	Metformin (200 mg/kg)
Terminal weight (g)	188.40 ± 6.23^a^	225.50 ± 4.45^b^	200.60 ± 5.41^a^	185.60 ± 2.91^a^	199.33 ± 4.72^a^
Body weight gain (g)	46.00 ± 3.65^a^	88.00 ± 7.13^c^	62.40 ± 5.69^b^	57.60 ± 3.08^ab^	72.33 ± 1.82^b^
Average weight of the two ovaries (g) (× 10)	0.50 ± 0.07^a^	0.70 ± 0.04^b^	0.58 ± 0.06^ab^	0.57 ± 0.06^ab^	0.61 ± 0.02^ab^

The values are reported as mean ± SEM (*n* = 6). The values are organized in ascending sequence, from a to e according to Duncan statistical method. If there are common letters between the comparison groups, it indicates a non-significant difference (*p* > 0.05)

### Serum Biochemical Assessment

The FSH concentration decreased significantly (*p* < 0.05), while LH, LH/FSH, estrogen, and testosterone were significantly elevated in the PCOS group compared to the control rats. In contrast, a significant decline in all the elevated hormones was shown in the LSSE groups in a dose-dependent manner, as was the case with metformin compared to the PCOS group. At the same time, the FSH significantly increased in the LSSE (500 mg/kg) group only (Table 3).

**Table 3:** The effect of LSSE on the hormonal concentration of PCOS rats

	Control	PCOS	LSSE (250 mg/kg)	LSSE (500 mg/kg)	Metformin (200 mg/kg)
FSH (mU/ml)	0.68 ± 0.03^c^	0.32 ± 0.01^a^	0.34 ± 0.02^a^	0.39 ± 0.02^b^	0.33 ± 0.01^a^
LH (mU/ml)	0.14 ± 0.01^a^	1.74 ± 0.09^d^	0.54 ± 0.04^c^	0.35 ± 0.02^b^	0.47 ± 0.02^bc^
LH: FSH ratio	0.21 ± 0.01^a^	5.41 ± 0.15^d^	1.60 ± 0.08^c^	0.88 ± 0.03^b^	1.42 ± 0.04^c^
Estrogen (pg/ml)	17.53 ± 1.19 ^a^	37.14 ± 1.89^d^	25.09 ± 0.87^c^	20.25 ± 1.21^ab^	22.96 ± 0.62 ^bc^
Testosterone (ng/ml)	0.27 ± 0.01^a^	3.07 ± 0.09^e^	1.01 ± 0.06^c^	0.51 ± 0.01^b^	1.20 ± 0.04^d^

The values are reported as mean ± SEM (*n* = 6). The values are organized in ascending sequence, from a to e according to Duncan statistical method. If there are common letters between the comparison groups, it indicates a non-significant difference (*p* > 0.05)

It was found that the PCOS group had significantly higher (*p* < 0.05) glucose, ALT, AST, ALP, total cholesterol, and LDL, while the HDL was significantly lower compared to the control group. LSSE (500 mg/kg) and metformin, on the other hand, were able to lower glucose significantly (*p* < 0.05). Also, LSSE and metformin treatment reduced ALT, AST, ALP, total cholesterol and LDL levels compared to the PCOS group (Table 4).

**Table 4:** The effect of LSSE on glucose concentration, liver function markers and lipid profile of PCOS rats

	Control	PCOS	LSSE (250 mg/kg)	LSSE (500 mg/kg)	Metformin (200 mg/kg)
Glucose (mg/dl)	102.50 ± 2.03 ^a^	138.79 ± 1.65^c^	133.7 ± 3.33^c^	109.14 ± 2.33 ^ab^	115.56 ± 2.05^b^
ALT (U/L)	47.28 ± 1.87^a^	88.06 ± 2.45^d^	61.44 ± 1.41^bc^	56.13 ± 1.94^b^	63.61 ± 3.55^c^
AST (U/L)	80.34 ± 2.37^a^	179.84 ± 4.52^d^	113.14 ± 1.46^c^	99.34 ± 2.24^b^	104.26 ± 2.74^b^
ALP (U/L)	255.20 ± 9.27^a^	953.66 ± 40.17^d^	368.52 ± 8.92^c^	300.06 ± 18. 99^ab^	336.63 ± 3.78^bc^
Total proteins (g/dl)	5.39 ± 0.05^a^	5.53 ± 0.20^a^	4.83 ± 0.53^a^	4.64 ± 0.25^a^	4.50 ± 0.20^a^
Albumin (g/dl)	3.41 ± 0.08^a^	3.41 ± 0.07^a^	3.56 ± 0.14^a^	3.25 ± 0.19^a^	3.47 ± 0.19^a^
Total cholesterol (mg/dl)	80.55 ± 4.82 ^a^	121.34 ± 3.81^b^	115.11 ± 4.57^b^	90.67 ± 2.88^a^	119.74 ± 2.93^b^
LDL (mg/dl)	40.01 ± 2.08^a^	53.57 ± 2.41 ^b^	49.56 ± 2.05^b^	42.06 ± 2.28^a^	50.25 ± 1.06^b^
HDL (mg/dl)	59.35 ± 2.36^b^	40.99 ± 2.04^a^	43.57 ± 2.75 ^a^	46.34 ± 1.30^a^	44.75 ± 2.01^a^

The values are reported as mean ± SEM (*n* = 6). The values are organized in ascending sequence, from a to e according to Duncan statistical method. If there are common letters between the comparison groups, it indicates a non-significant difference (*p* > 0.05)

The kidney profile markers (creatinine, urea and uric acid) and LDH activity increased significantly (*p* < 0.05) in the PCOS group compared to the control group. At the same time, the mineral concentration of calcium and phosphorus in serum declined. Meanwhile, treatment with LSSE and metformin significantly declined all these markers in a dose-dependent manner compared to the PCOS group. Creatinine showed a decrease after treatment with LSSE (500 mg/kg) and metformin only. On the contrary, rats treated with LSSE or metformin caused a significant elevation in mineral concentration in a dose-dependent manner (Table 5).

**Table 5:** The effect of LSSE on kidney function markers, LDH activity and minerals concentration of PCOS rats

	Control	PCOS	LSSE (250 mg/kg)	LSSE (500 mg/kg)	Metformin (200 mg/kg)
Creatinine (mg/dl)	0.39 ± 0.01^a^	0.82 ± 0.02^c^	0.75 ± 0.02^c^	0.59 ± 0.04^b^	0.59 ± 0.04^b^
Urea (mg/dl)	29.09 ± 1.34^a^	54.70 ± 4.71^c^	34.78 ± 2.56^ab^	37.55 ± 2.97^ab^	41.71 ± 3.02^b^
Uric acid (mg/dl)	1.65 ± 0.14^a^	4.01 ± 0.23^c^	2.27 ± 0.05^b^	2.06 ± 0.08^ab^	2.08 ± 0.21^ab^
LDH (U/L)	262.12 ± 10.69^a^	1941.45 ± 72.11^d^	835.62 ± 17.18^c^	666.05 ± 16.51^b^	675.09 ± 15.14^b^
Calcium (mg/dl)	34.60 ± 0.76^d^	4.89 ± 0.28^a^	17.13 ± 0.54^b^	25.93 ± 1.55^c^	23.76 ± 0.97^c^
Phosphorous (mg/dl)	6.86 ± 0.35^c^	4.54 ± 0.32^a^	5.64 ± 0.23^b^	6.47 ± 0.33^c^	5.44 ± 0.12^b^

The values are reported as mean ± SEM (*n* = 6). The values are organized in ascending sequence, from a to e according to Duncan statistical method. If there are common letters between the comparison groups, it indicates a non-significant difference (*p* > 0.05)

### Oxidative Stress Markers

The present data illustrated that PCOS significantly increased (*p* < 0.05) MDA and NO values in the ovarian and uterine tissues compared to the control. On the contrary, the values of GSH and CAT were decreased. These changes in the oxidative parameters were reversed after treatment with LSSE and metformin, which significantly reduced the MDA and NO while elevating the GSH and CAT (Table 6).

**Table 6:** The effect of LSSE on the oxidative stress markers in the ovarian and uterine tissues of PCOS rats

		Control	PCOS	LSSE (250 mg/kg)	LSSE (500 mg/kg)	Metformin (200 mg/kg)
MDA (nmol/g.tissue)	Ovary	7.73 ± 0.20^a^	13.36 ± 0.40^d^	11.10 ± 0.26^c^	8.84 ± 0.18^b^	9.42 ± 0.18^b^
GSH (mmol/g.tissue)		1.15 ± 0.07^c^	0.59 ± 0.05^a^	0.91 ± 0.04^b^	1.02 ± 0.05^bc^	1.07 ± 0.04^bc^
CAT (U/g.tissue)		56.95 ± 1.81^e^	22.72 ± 0.49^a^	26.70 ± 0.47^b^	33.47 ± 0.50 ^d^	30.33 ± 1.27^c^
NO (µmol/g.tissue)		790.30 ± 32.45^a^	2125.48 ± 57.55^d^	1501.65 ± 44.08^c^	1323.28 ± 41.74^b^	1592.30 ± 26.88^c^
MDA (nmol/g.tissue)	Uterus	4.83 ± 0.14^a^	7.85 ± 0.13^c^	7.21 ± 0.07^b^	6.85 ± 0.07^b^	6.97 ± 0.20^b^
GSH (mmol/g.tissue)		2.23 ± 0.03^e^	1.01 ± 0.06^a^	1.44 ± 0.02^c^	1.70 ± 0.01^d^	1.19 ± 0.03^b^
CAT (U/g.tissue)		58.43 ± 1.05^d^	28.27 ± 1.00^a^	30.73 ± 0.84^a^	46.25 ± 2.10^c^	36.78 ± 1.26^b^
NO (µmol/g.tissue)		609.32 ± 16.83^a^	1284.12 ± 52.84^d^	1024.87 ± 25.64^c^	856.58 ± 38.20^b^	805.88 ± 21.65^b^

The values are reported as mean ± SEM (*n* = 6). The values are organized in ascending sequence, from a to e according to Duncan statistical method. If there are common letters between the comparison groups, it indicates a non-significant difference (*p* > 0.05)

### DNA Fragmentation

The PCOS rats showed a significant surge (p < 0.05) in both tissues compared to the control rats. On the other hand, LSSE and metformin treatments could significantly lower this fragmentation in a dose-dependent manner compared to the PCOS rats (Table 7).

**Table 7:** The effect of LSSE on the DNA fragmentation concentration in the ovarian and uterine tissues of PCOS rats

		Control	PCOS	LSSE (250 mg/kg)	LSSE (500 mg/kg)	Metformin (200 mg/kg)
Fragmented DNA (ng/ml)	Ovary	0.33 ± 0.05^a^	3.87 ± 0.16^c^	1.68 ± 0.10^b^	1.50 ± 0.09^b^	1.63 ± 0.04^b^
	Uterus	307.33 ± 30.53^a^	2391.67 ± 80.21^c^	2116.67 ± 120.52^c^	1516.33 ± 10.84^b^	2298.67 ± 204.04^c^

The values are reported as mean ± SEM (*n* = 6). The values are organized in ascending sequence, from a to e according to Duncan statistical method. If there are common letters between the comparison groups, it indicates a non-significant difference (*p* > 0.05)

### Histopathological Examination

#### Histopathological Examination of the Ovary

**Fig. 2:**
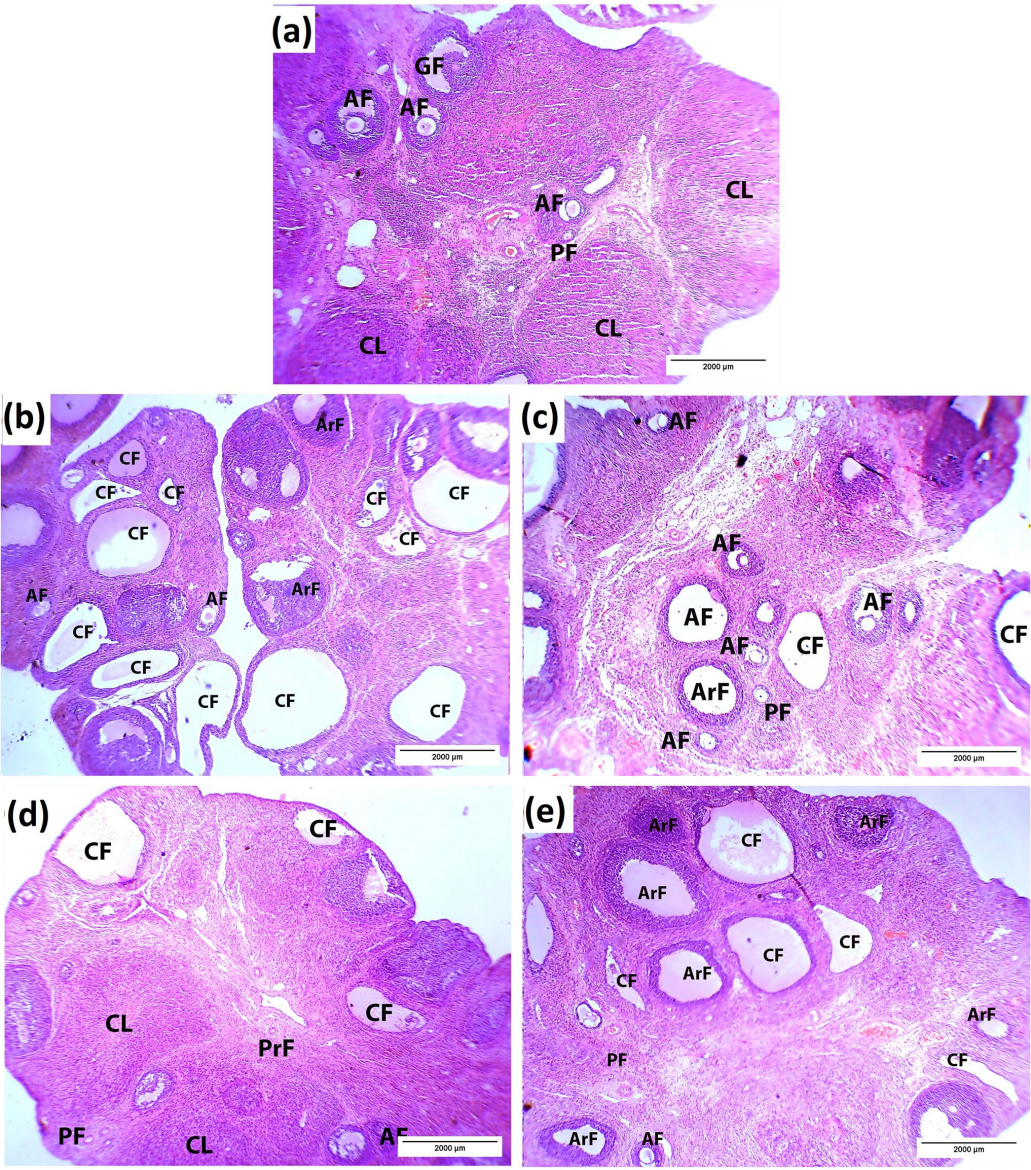
Photomicrographs showing the effect of LSSE on the histopathological alterations in the ovary tissue of rats of different groups (H&E). (**a)** control group, (**b**) PCOS group, (**c**) LSSE (250 mg/kg) group, (**d**) LSSE (500 mg/kg) group, and (**e**) metformin group. PF: primary follicles, AF: antral follicles, GF: Graafian follicles, CL: corpora lutea, CF: cystic follicles, and ArF: atretic follicles

Histological assessment revealed that ovarian sections of the control group showed typical tissue structure. The ovary had an outer cortex and inner medulla. The peripheral cortex had several developing follicles. Unilaminar primary follicles are small, with a single layer of flattened granulosa cells surrounding the oocyte. Multilaminar primary follicles are slightly larger with multiple layers. Antral follicles are large, with a fluid-filled antral cavity, numerous granulosa layers, and a distinct theca layer. Centrally positioned medulla included loose connective tissue and blood vessels. The organization follows normal folliculogenesis in a healthy rat ovary (Fig. 2a).

The PCOS group showed apparent histopathological alterations, such as severe follicular atresia; fewer normal follicles, many large cystic follicles, and some atretic follicles. The large follicular cysts were scattered in both the medulla and cortex and were characterized by a thin layer of flat cells resting on a fibrous membrane and an absent oocyte. On the other hand, the atretic follicles had a thick granulosa wall and no oocyte (Fig. 2b). The number of antral follicles and corpora lutea was significantly decreased (p < 0.05) in the PCOS group, while atretic and cystic follicles were increased (Table 8).

**Table 8:** The effect of LSSE on the scoring system of the ovarian follicles, cystic follicle diameter and uterine tissue (/ section)

		Control	PCOS	LSSE (250 mg/kg)	LSSE (500 mg/kg)	Metformin (200 mg/kg)
Antral follicles	Ovary	6.00 ± 0.29^b^	4.44 ± 0.34^a^	5.44 ± 0.44^ab^	5.56 ± 0.38^ab^	5.44 ± 0.50^ab^
Graafian follicles		2.00 ± 0.29^b^	1.67 ± 0.17^ab^	1.67 ± 0.17^ab^	1.44 ± 0.18^ab^	1.33 ± 0.17^a^
Corpora lutea		7.78 ± 1.42^b^	3.67 ± 0.29^a^	3.89 ± 0.20^a^	4.33 ± 0.29^a^	3.56 ± 0.84^a^
Atretic follicles		10.78 ± 0.98^b^	18.4 ± 0.99^c^	8.00 ± 0.44^a^	9.00 ± 0.50^ab^	9.22 ± 0.40^ab^
Cystic follicles (CF)		0.00 ± 0.00^a^	14.0 ± 1.90^c^	11.22 ± 1.52^bc^	9.56 ± 0.75^b^	9.78 ± 0.57^b^
CF diameter (mm)		-	1.15 ± 0.19^b^	0.80 ± 0.01^b^	0.72 ± 0.01^a^	1.11 ± 0.08^b^
Thickness of wall (μm)	Uterus	389.21 ± 37.84^b^	247.6 ± 12.91^a^	216.90 ± 79.04^a^	274.65 ± 11.23^a^	232.64 ± 16.15^a^
No. of endometrial glands		12.33 ± 0.33^b^	8.89 ± 0.84^a^	11.00 ± 1.31^ab^	10.89 ± 0.26^ab^	10.89 ± 0.70^ab^

The values are reported as mean ± SEM. The values are organized in ascending sequence, from a to e according to Duncan statistical method. If there are common letters between the comparison groups, it indicates a non-significant difference (*p* > 0.05)

The LSSE (250 mg/kg)-treated rats had better histopathology than the PCOS group. The ovary showed some primary and antral follicles and fewer small cystic and atretic follicles than the PCOS group (Fig. 2c). Moreover, the number of the antral, Graafian, and cystic follicles, as well as the corpora lutea, and average cyst size in the ovary of the LSSE (250 mg/kg) group changed insignificantly compared to the PCOS group. On the other hand, the number of atretic follicles was significantly reduced (Table 8).

The group treated with LSSE (500 mg/kg) had more enhanced ovarian histoarchitecture, showing multiple primordial, primary, antral follicles and a few little cystic follicles (Fig. 2d). Although the antral, Graafian follicles and corpora lutea counts changed insignificantly in the LSSE high-dose group compared to the PCOS group, there was a significant reduction in the number of both atretic and cystic follicles and their size (Table 8).

The metformin-treated group showed improved ovarian histomorphology, as the ovary shows numerous primary and antral follicles and a few medium-sized cystic and atretic follicles (Fig. 2e). However, the number of the normal developing follicles changed insignificantly (*p* < 0.05) compared to the PCOS group, while the cystic and atretic follicle were significantly less but almost the same size (Table 8).

**Fig. 3:**
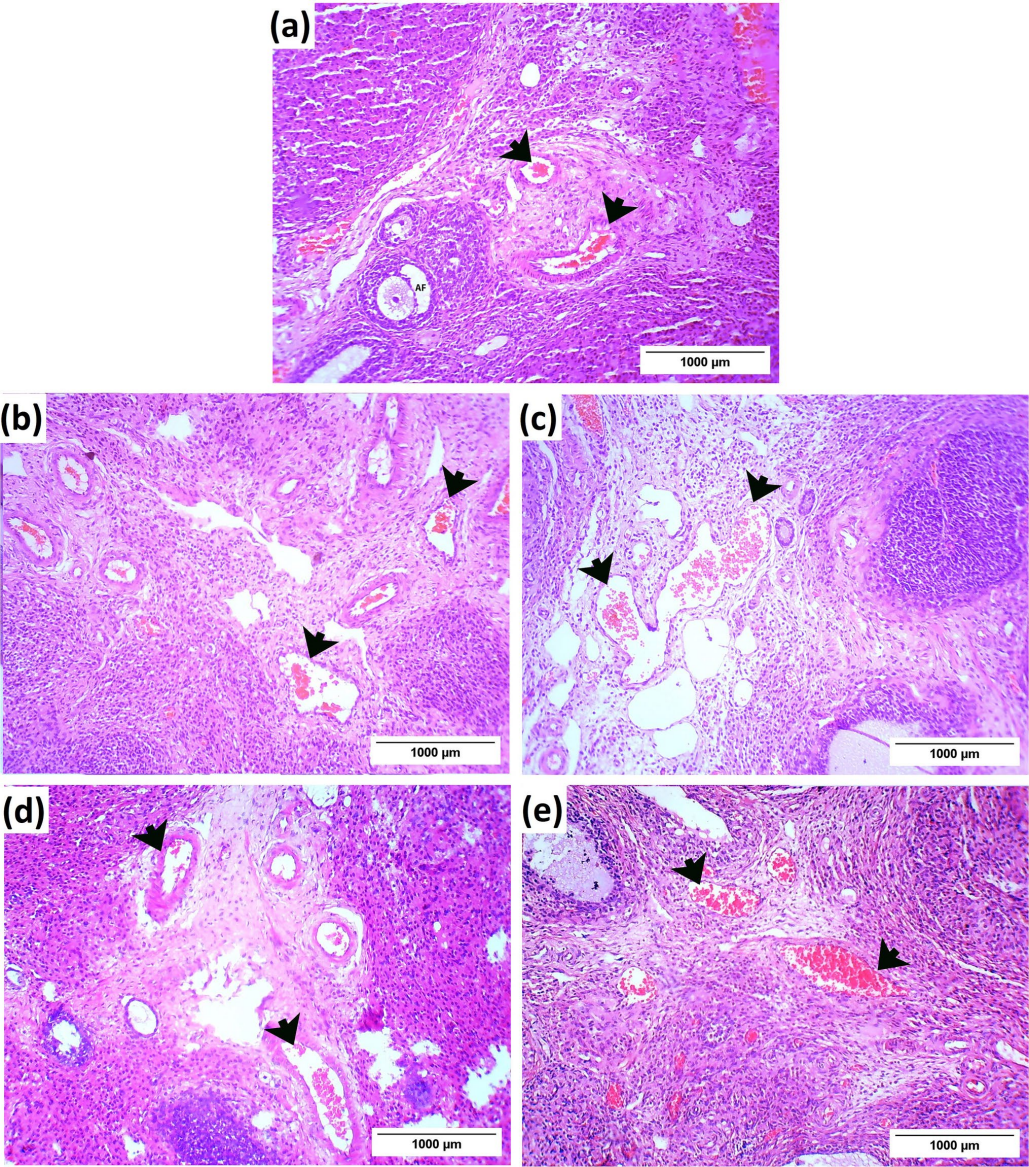
Photomicrographs showing the effect of LSSE on the blood vessels morphology in the medulla of the ovarian tissue (H&E). (**a)** control group, (**b**) PCOS group, (**c**) LSSE (250 mg/kg) group, (**d**) LSSE (500 mg/kg) group, and (**e**) metformin group. Black arrow: blood vessel

Regarding the blood vessels, the control group showed that the medulla displayed highly vascularized connective tissue with normal blood vessels (Fig. 3a). Controversially, dilation and congestion of the interstitial blood capillaries were prevalent in the medulla of the PCOS group (Fig. 3b). Also, the LSSE (250 mg/kg) group showed enormously dilated blood vessels with mild congestion (Fig. 3c). Meanwhile, the LSSE (500 mg/kg) group showed mildly dilated blood vessels with no congestion, while the metformin-treated group showed mildly dilated blood vessels with solid congestion (Fig. 3d and e).

**Fig. 4:**
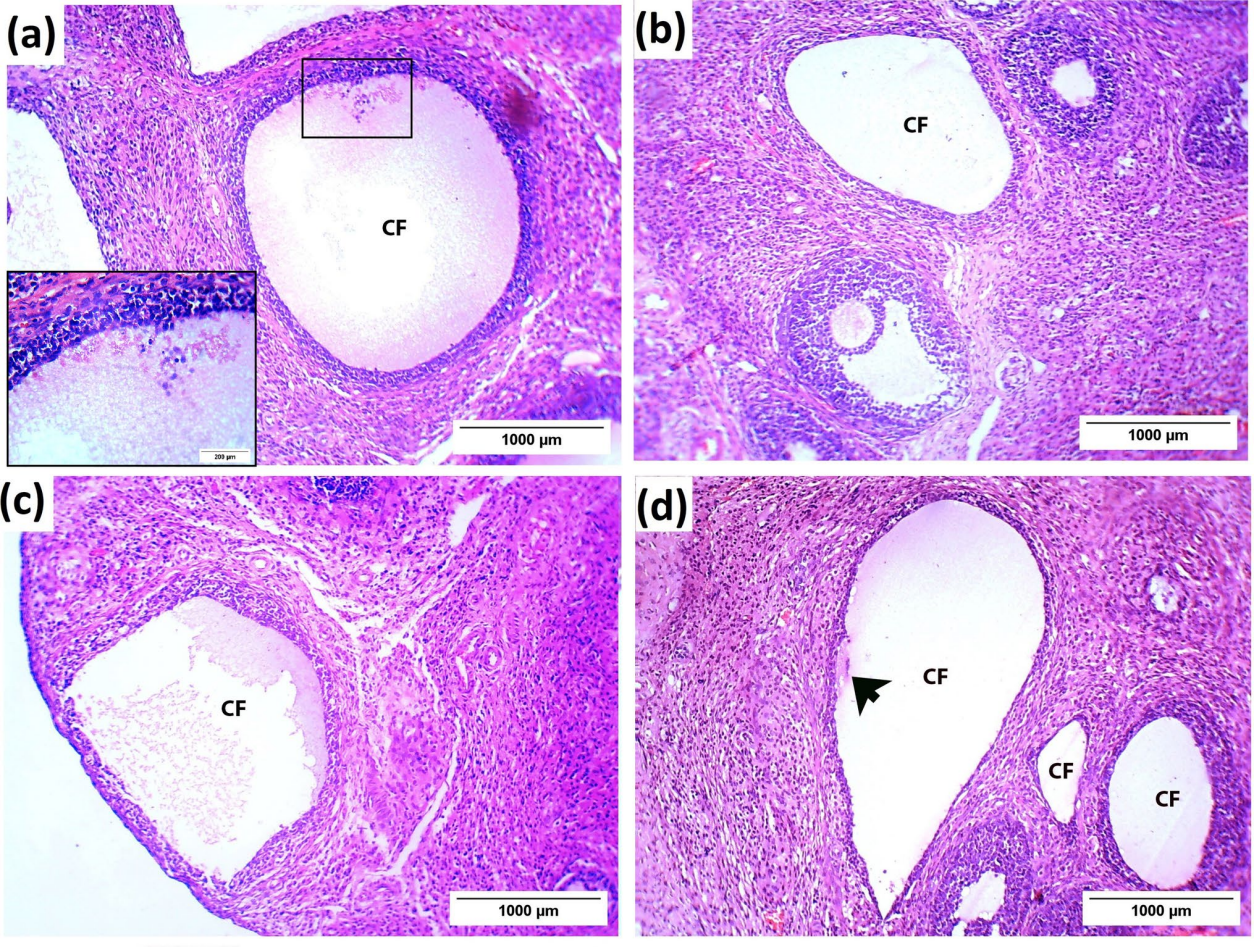
Photomicrographs showing the effect of LSSE on the cystic follicle morphology in the ovarian tissue of PCOS rats (H&E). (**a)** PCOS group, (**b**) (LSSE 250 mg/kg) group, (**c**) LSSE (500 mg/kg) group, and (**d**) metformin group. CF: cystic follicle and black arrow: detached granulosa cells

The PCOS group is mainly characterized histologically by the cystic follicle lacking an oocyte, decaying granulosa cells, vacuolated cytoplasm, and shrunken, darkly pigmented pyknotic nuclei detached in the antral cavities (Fig. 4a). Meanwhile, the groups treated with LSSE showed a smaller thin-walled cystic follicle than the PCOS group and didn't show detached granulosa cells (Fig. 4b and c). On the contrary, the metformin-treated group showed a sheet of detached granulosa in the antral cavity of the cystic follicle. It was closer in size to the PCOS group (Fig. 4d).

**Fig. 5:**
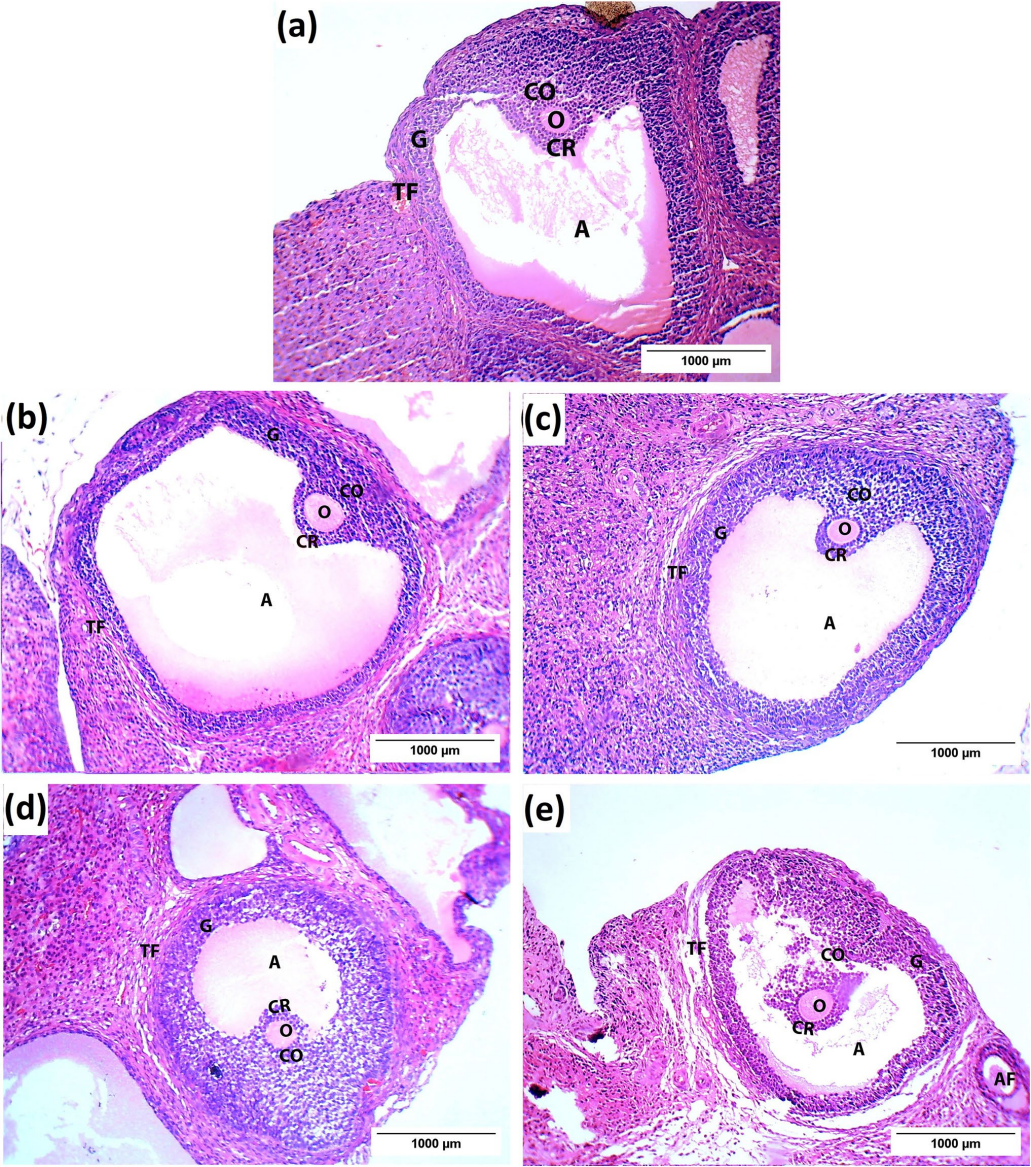
Photomicrographs showing the effect of LSSE on the Graafian follicles of the rat ovary (H&E). (**a**) control group, (**b**) PCOS group, (**c**) LSSE (250 mg/kg), (**d**) LSSE (500 mg/kg), and (**e**) metformin group. A: antrum, CR: corona radiata, CO: cumulus oophorus, TF: theca folliculi and G: granulosa wall

The Graafian follicle didn't show many alterations between the different groups. It was evident in all groups as a distinctive large fluid-filled antrum with the oocyte encircled by corona radiata and cumulus oophorus connecting to the granulosa wall of cells forming the follicle and all surrounded by the theca folliculi (Fig. 5a). The PCOS group has an exception where it exhibited a thinner wall of granulosa cells (Fig. 5b). Although the LSSE groups showed normal Graafian follicles, the metformin-treated group showed a partially degenerated cumulus oophorus, and the oocyte was partially detached from the wall (Fig. 5c-e).

#### Histopathological Examination of the Uterine Tissue

**Fig. 6:**
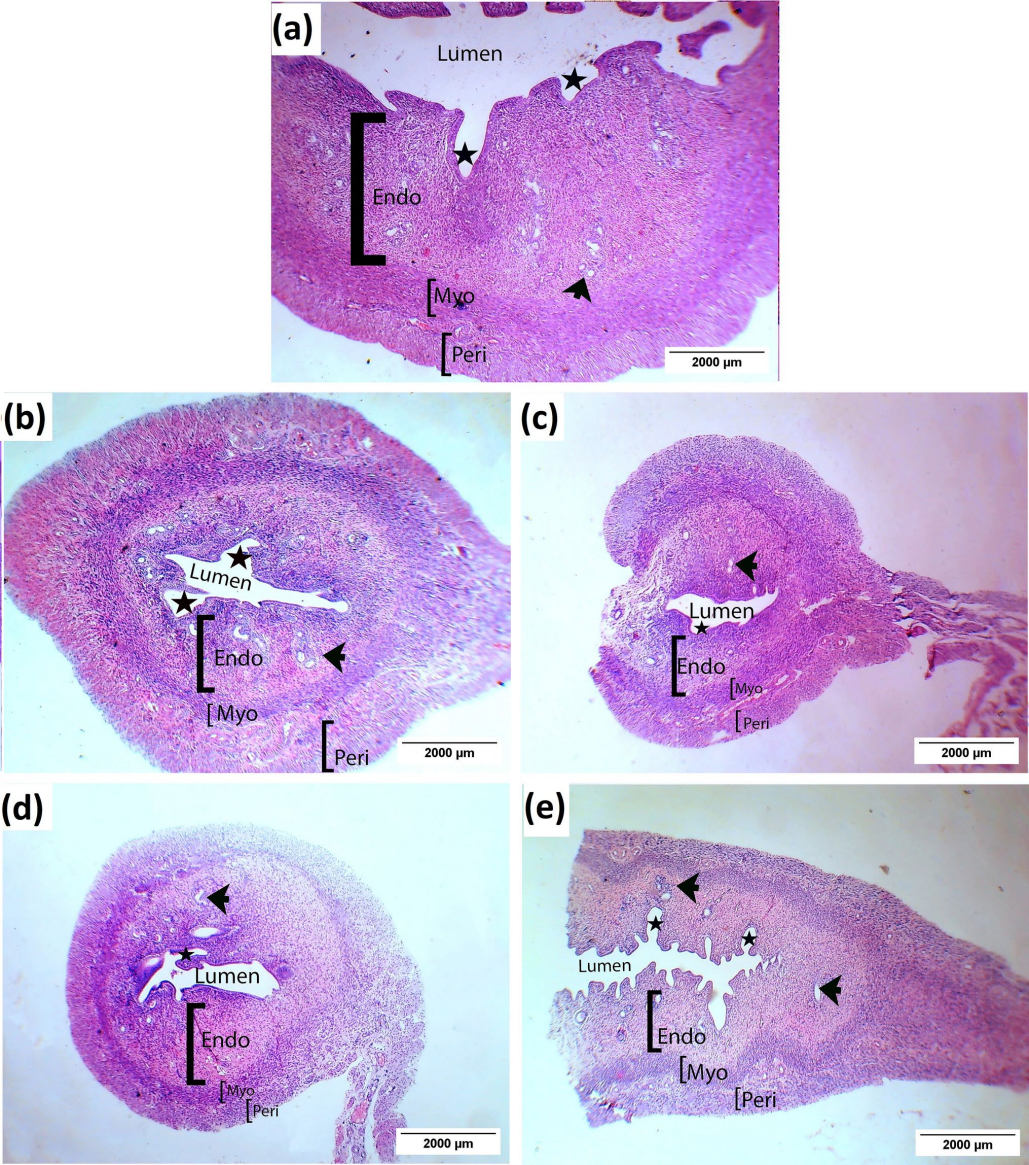
Photomicrographs showing the effect of LSSE on the morphology of the uterine tissue of the PCOS rats (H&E (**a**) control group, (**b**) PCOS group, (**c**) LSSE (250 mg/kg) group, (**d**) LSSE (500 mg/kg) and (**e**) metformin group. Endo: Endometrium, Myo: myometrium, Peri: perimetrium, star: mucosal fold, and black arrow: endometrial gland

The uterine tissue sections of the control group had normal histoarchitecture. They showed the three layers that are typical of uterine histology: the endometrium (innermost layer), the myometrium (middle layer), and the perimetrium (outermost layer). The endometrium indicated a healthy uterine environment, with a thick and wide inner uterine lumen and multiple mucosal folds. Many endometrial glands and a thick uterine wall were observed within the endometrium (Table 8, Fig. 6a).

Inversely, the examined uterine tissue sections of the PCOS rats group displayed an alteration in the histomorphology, which revealed increased thickness of the perimetrium layer, reduced thickness in the endometrium, narrowing of the uterine lumen, and fewer mucosal folds compared to the control group. In addition, the number of uterine glands and the thickness of the uterine wall were significantly reduced compared to the control group (*p* < 0.05) (Table 8, Fig. 6b).

The LSSE (250 mg/kg)-treated group displayed a narrow lumen, endometrium, and perimetrium, fewer mucosal folds, and average thickness myometrium compared to the PCOS group. The uterus showed an insignificantly thinner (*p* > 0.05) uterine wall compared to the PCOS group (Table 8, Fig. 6c).

The LSSE (500 mg/kg)-treated group exhibited a wider lumen, more mucosal folds, and a thicker endometrium than the PCOS group, while the myometrium and perimetrium displayed average thickness. However, the uterine wall was insignificantly thicker than the PCOS group (Table 8, Fig. 6d).

The metformin-treated group showed average myometrium and perimetrium thickness, while the endometrial thickness was thicker, and the lumen had more mucosal folds than the PCOS group. However, the uterine wall was insignificantly thinner than the PCOS (Table 8, Fig. 6e).

Moreover, all the treated groups showed a nonsignificant change (*p* > 0.05) in the number of endometrial glands compared to the PCOS group (Table 8).

### Evaluation of Tumor Necrosis Factor-Alpha (TNF- α)

#### Tumor Necrosis Factor-Alpha (TNF-α) of the Ovarian Tissue

**Fig. 7:**
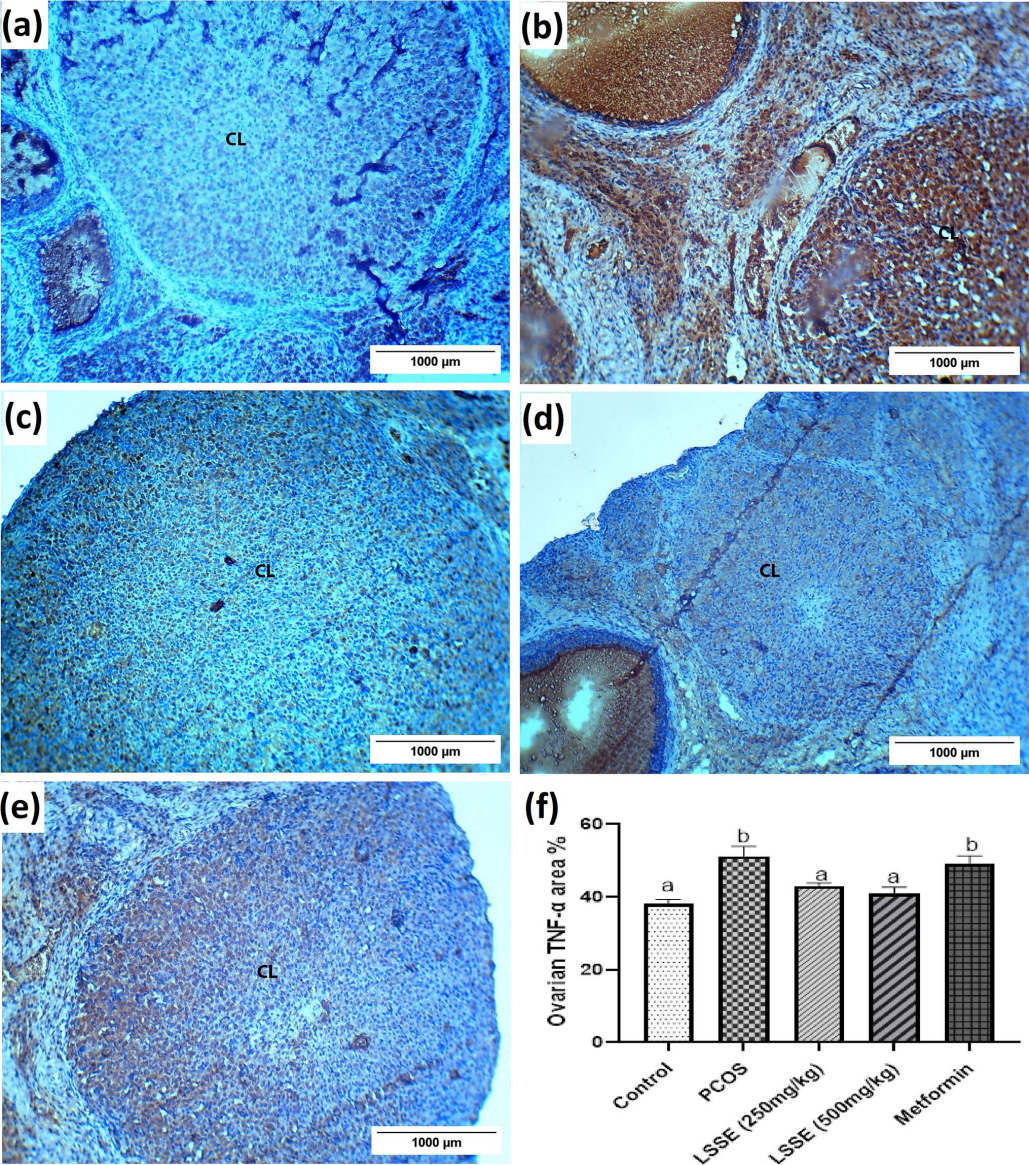
Photomicrographs showing the effect of LSSE on the immunohistochemistry of TNF- α expression in ovarian tissue of the corpus luteum in PCOS rats. (**a**) control group, (**b**) PCOS group, (**c**) LSSE (250 mg/kg) group, (**d**) LSSE (500 mg/kg) group, (**e**) metformin (200 mg/kg) group and (**f**) The histogram showing the effect of LSSE on the immunohistochemistry of TNF- α expression in ovarian tissue of PCOS rats. The values are organized in ascending sequence, from a to e according to Duncan statistical method. If there are common letters between the comparison groups, it indicates a non-significant difference (*p* > 0.05).CL: corpus luteum

The ovary of the control group showed weak TNF-α immunoreactivity in luteal cells (Fig. 7a). On the contrary, the ovary of the PCOS group showed excessive immunoreactivity in luteal and interstitial cells (Fig. 7b). However, the LSSE groups' ovaries showed weak TNF-α immunoreactivity, while the metformin showed moderate TNF-α immunoreactivity (Fig. 7c-e). The ovarian tissue's TNF-α area % of the PCOS group increased significantly (*p* < 0.05) compared to the control group. However, a significant reduction in the ovarian TNF-α area % was measured after administering LSSE, where both groups showed negative immunoreactions. In contrast, the metformin group was insignificantly changed compared to the PCOS group (Fig. 7f).

#### Tumor Necrosis Factor-Alpha (TNF-α) in the Uterine Tissue

**Fig. 8:**
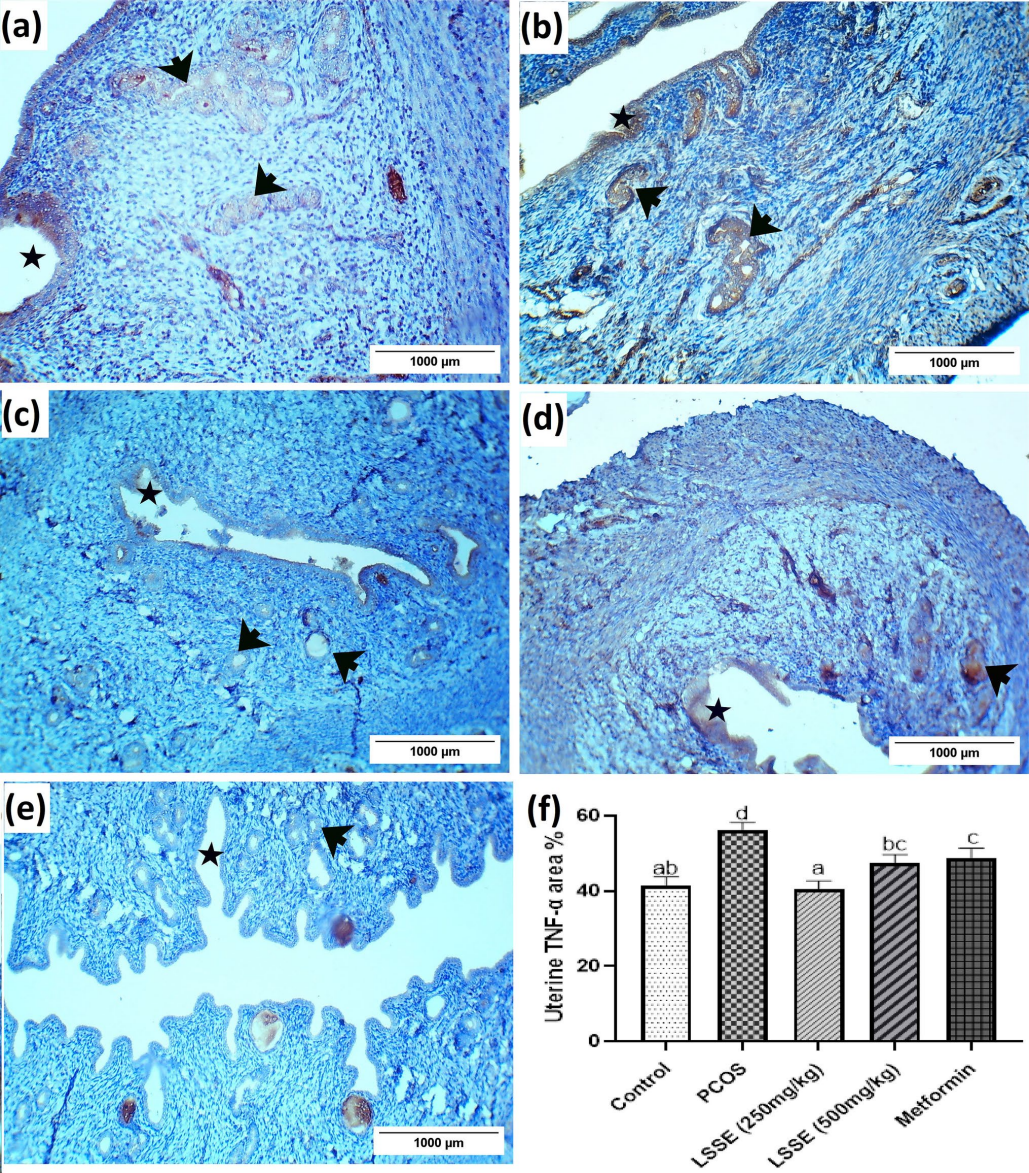
Photomicrographs showing the effect of LSSE on the immunohistochemistry of TNF- α expression in uterine tissue of the columnar epithelial cells of mucosal folds of the lumen and endometrial glands in PCOS rats. (**a**) control group, (**b**) PCOS group, (**c**) LSSE (250 mg/kg) group, (**d**) LSSE (500 mg/kg) group, (**e**) metformin (200 mg/kg) group and (**f**) histogram showing the effect of LSSE on the area percentage % of TNF- α immuno-expression in uterine tissues of PCOS rats. The values are organized in ascending sequence, from a to e according to Duncan statistical method. If there are common letters between the comparison groups, it indicates a non-significant difference (*p* > 0.05). Star: mucosal folds of the lumen and black arrow: endometrial glands

The uterus of the PCOS group showed excessive TNF-α immunoreactivity in epithelial cells lining the lumen and endometrial glands (Fig. 8b) compared to the control (Fig. 8a). However, the LSSE (250 mg/kg) group's uterus showed weak immunoreactivity in cells lining the lumen and endometrial glands (Fig. 8c). On the other hand, the LSSE (500 mg/kg) group showed moderate immunoreactivity (Fig. 8d). Additionally, the uterus of the metformin group showed negative TNF-α immunoreactivity in the cells of the lumen and weakness in the endometrial glands (Fig. 8e). The TNF-α area % in the uterine tissue of the PCOS group significantly increased (*p*<0.05) compared to the control group. On the contrary, The LSSE (250 mg/kg) group showed negative immunoreactivity, while the LSSE (500 mg/kg) and metformin groups showed significantly lower immunoreactivity than the PCOS group (Fig. 8f).

### Female Fertility Assessment

The PCOS rats revealed lower mating and fertility indices than the control group. On the other hand, LSSE (500 mg/kg) and metformin enhanced the indices compared to the PCOS group (Table 9).

**Table 9:** The effect of LSSE on the mating and fertility indices %, maternal body weight, pregnancy outcomes and morphological examination of the fetuses of PCOS rats.after mating trials with normal males

	Control	PCOS	LSSE (250 mg/kg)	LSSE (500 mg/kg)	Metformin (200 mg/kg)
Mating and fertility indices					
Mating index ^a^ (%)	100.00	50.00	50.00	83.33	83.33
Fertility index ^b^ (%)	100.00	66.67	66.67	80.00	80.00
Maternal parameters					
Maternal terminal body weight (g)	225.33 ± 7.26^a^	221.50 ± 21.50^a^	247.40 ± 10.93^a^	223.67 ± 10.93^a^	257.25 ± 7.20^b^
No. Corpora Lutea /litter	8.17 ± 0.70^a^	10.50 ± 2.50^ab^	11.50 ± 0.50^b^	9.25 ± 0.48^ab^	9.00 ± 0.4^ab^
No. implantation sites /litter	8.00 ± 0.77^a^	5.50 ± 0.50^a^	8.50 ± 0.50^a^	6.25 ± 1.03^a^	7.25 ± 0.95^a^
Preimplantation loss index %	2.33 ± 2.33^a^	46.00 ± 8.00^c^	26.00 ± 1.00^ab^	32.75 ± 9.88^bc^	19.75 ± 8.10^ab^
Postimplantation loss index %	0.00 ± 0.00^a^	8.34 ± 8.34 ^a^	0.00 ± 0.00^a^	8.33 ± 8.33^a^	2.50 ± 2.50^a^
Gravid uterine weight (g)	32.14 ± 1.88^bc^	24.55 ± 1.45^ab^	40.60 ± 3.60^c^	21.67 ± 2.73^a^	29.63 ± 3.29^ab^
Placenta weight (g)	0.53 ± 0.02^a^	0.61 ± 0.03^a^	0.58 ± 0.01^a^	0.59 ± 0.01^a^	0.60 ± 0.06^a^
Fetal examination					
No. live fetuses/litter	8.00 ± 0.77^ab^	5.00 ± 0.00^a^	8.50 ± 0.50^b^	5.75 ± 1.18^ab^	7.00 ± 0.71^ab^
No. dead fetuses/litter	0.00 ± 0.00^a^	0.50 ± 0.50^b^	0.00 ± 0.00^a^	0.00 ± 0.00^a^	0.00 ± 0.00^a^
Body weight of fetuses(g)	2.35 ± 0.15^a^	2.23 ± 0.21^a^	2.52 ± 0.00^a^	2.49 ± 0.14^a^	2.21 ± 0.24^a^
External abnormalities/litter (Hematoma)	0.00 ± 0.00^a^	0.50 ± 0.50^a^	0.00 ± 0.00^a^	0.00 ± 0.00^a^	0.25 ± 0.25^a^

The values are reported as mean ± SEM. The values are organized in ascending sequence, from a to e according to Duncan statistical method. If there are common letters between the comparison groups, it indicates a non-significant difference (*p* > 0.05)

a: $$\frac{\text{Number of females with successful copulation}}{\text{Number of females cohabited}}\text{x }100$$ b:$$\frac{\text{Number of pregnant females}}{\text{Number of mated female}}\text{x }100$$

### Maternal Toxicity Assessment

All dams of different groups throughout gestational periods showed no deaths or signs of illness or abnormal behavior except decreased activity.

### Analysis of Pregnancy Outcomes

**Fig. 9:**
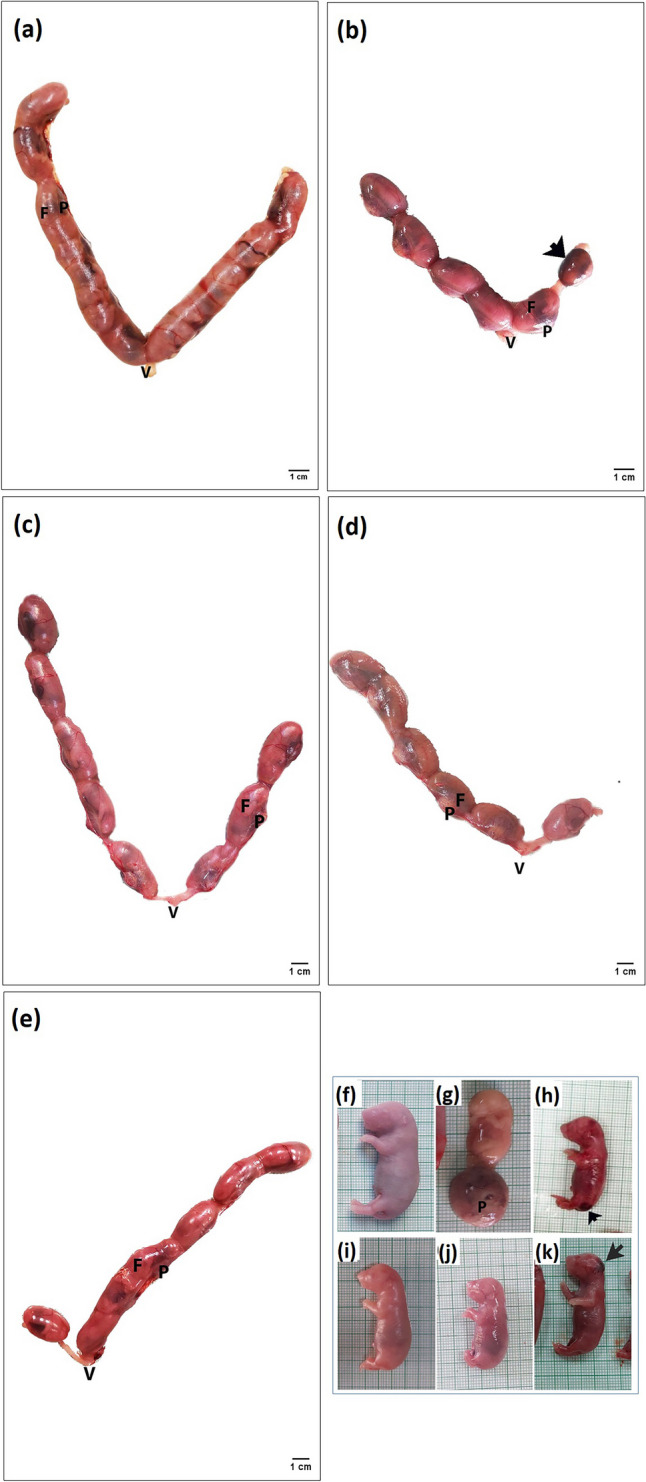
Photographs showing the effect of LSSE on the morphology of the uterus and fetuses of the PCOS pregnant rats on the 20th day of pregnancy. (**a**) control group, (**b**) PCOS group, (**c**) LSSE (250 mg/kg) group, and (**d**, **e**) LSSE (500 mg/kg) and metformin (200 mg/kg) groups. (**f**): control group, (**g** & **h**) PCOS group, (**i** & **j**) LSSE (250 mg/kg) and LSSE (500 mg/kg) groups, and (**k**) metformin (200mg/kg) group. V: vagina, F: fetus, P: placenta

The number of the corpora lutea, implantation sites, postimplantation loss index, maternal terminal body weight, gravid uterine and placental weights, number and body weight of fetuses changed insignificantly (*p* > 0.05) between all groups (Table 9). However, the preimplantation loss was elevated significantly (*p *< 0.05) in the PCOS group compared to the control group and exhibited a significant decline in the LSSE (250 mg/kg) and metformin groups compared to the PCOS group (Table 9). Meanwhile, the number of corpora lutea, gravid uterine weight, and live fetuses significantly elevated (*p* < 0.05) in the LSSE (250 mg/kg) group compared to the PCOS group (Table 9). On the other hand, the metformin group showed an exceptional increase in maternal body weight compared to all groups (Table 9). Additionally, one dead fetus was found in the PCOS group only, while control and treated mothers didn't have any dead fetuses (Fig. 9 g). In addition, no signs of external abnormalities were recorded, except in fetuses whose mothers had untreated PCOS or were treated with metformin (200 mg/kg), where they showed hematoma in different body parts (Table 9, Fig. 9 h and k).

The morphology of the gravid uterus showed some alternations between groups. The control group showed normal gross morphology and normally distributed embryos in both horns (Fig. 9a). Meanwhile, the PCOS, LSSE (500 mg/kg), and metformin groups showed an unequal distribution of fetuses, and the PCOS uterus showed a dead fetus (Fig. 9b). The LSSE (250 mg/kg) group showed a notable increase in the number of embryos and a normal distribution of embryos (Fig. 9c).

## Discussion

PCOS is a prevalent endocrine disorder affecting women of reproductive age, with a significant impact on fertility and pregnancy outcomes [26]. LSSE is a natural extract packed with beneficial phytochemicals and nutrients and has proved its efficacy against multiple diseases [27]. The effects of the LSSE on PCOS, fertility, and pregnancy outcomes haven't been investigated. Thus, the current study focused on the physiological and histological effects of one of the most common disorders on rats and their pregnancy outcomes after its treatment with the extract of natural seeds.

PCOS rats exhibited increased final body weight, average weight gain, and ovarian weight compared to control rats. These changes were associated with elevated glucose, likely due to the hyperinsulinemic state and increased intra-abdominal fat deposition linked to hyperandrogenism [28]. Meanwhile, the increased ovarian weight is due to fluid-filled follicular cysts [29].

However, treatment with LSSE showed reduced terminal weight, average body weight gain, and glucose compared to the PCOS group. This effect is attributed to its insulin-sensitizing properties due to its quercetin and protocatechuic acid content [30].

Rats with PCOS exhibited hormonal imbalances characterized by elevated testosterone, LH, LH/FSH ratio, and estrogen alongside reduced FSH. This can be explained by the promotion of hyperinsulinemia to the testosterone production in ovarian theca cells causing LH/FSH equilibrium disturbance by increasing LH output and inhibiting FSH synthesis [31]**.** This elevated LH/ FSH ratio further stimulates testosterone production, creating a self-perpetuating cycle of hyperandrogenism [32]. The hyperinsulinemia resulting from insulin resistance also decreases hepatic production of sex hormone-binding globulin (SHBG), resulting in more free-circulating testosterone and estrogens [33]. Reactive oxygen species (ROS) and chronic inflammation greatly increase insulin resistance, adipose mass, and metabolic syndrome, leading to hormonal imbalance [34].

Conversely, LSSE therapy modulated this profile by elevating FSH and reducing testosterone, LH, the LH/FSH ratio, and estrogen in comparison to PCOS. Research has linked this regulation to the anthraquinone components of LSSE, which induce a transient modification in the feedback mechanism of the hypothalamic-pituitary–gonadal (HPG) axis by simulating the actions of endogenous estrogen [35]. LSSE's anti-diabetic characteristics reduce insulin, a crucial strategy for reducing androgens by directly blocking ovarian androgen synthesis via the LH theca route and enhancing hepatic SHBG production [36]. Also, lower insulin reduces cytochrome P450 17alpha-hydroxylase/17,20-lyas (CYP17) disruption, further decreasing androgen production [37]. As an insulin sensitizer, LSSE can reduce elevated anti-Mullerian hormone (AMH) production, restore regular ovulation, and regulate the HPG axis, thereby modulating LH/FSH concentrations [38].

This study revealed that the elevated glucose, liver and kidney function markers, and LDH in PCOS rats are due to the metabolic disturbances and dyslipidemia induced by hyperandrogenemia's disruption of lipid metabolism enzymes and insulin [39]. Hyperandrogenism, insulin resistance, and obesity promote fatty liver and inflammation, while excess lipid accumulation and ROS contribute to kidney damage and hepatocellular injury, causing enzyme leakage into circulation [40]. Also, these factors impair insulin signaling pathways, mainly through inhibiting glucose transporter type 4 (GLUT4) translocation, causing elevated glucose and excess adipocyte accumulation [41]. Despite these changes, normal total protein and albumin concentrations suggest that liver dysfunction may not be severe [42].

The current study revealed that LSSE effectively enhanced liver and kidney profiles, possibly due to its antioxidant and antidiuretic properties derived from phenols, alkaloids, and flavonoids [43]. Additionally, the kidney profile may be modulated by blocking tubular reabsorption of water and anions, boosting regional blood flow due to the LSSE's hepatoprotective kaempferol content [15, 44]. The lipid profile showed that LSSE reduced lipolysis, which reduced gluconeogenesis and insulin concentration [45]. Lipolysis regulation can promote weight loss by causing a reduction in the free fatty acids (FFA) that induce insulin sensitivity through activating phosphoinositide 3-kinase (PI3K) activity, which stimulates GLUT4 translocation [46]. Also, garden cress has a considerable amount of alpha-linolenic acid associated with decreased total cholesterol and LDL-TC [47].

This study measured significantly declined calcium and phosphorus concentrations in the serum of the PCOS rats. Insulin resistance is known to impair mineral homeostasis by affecting the regulatory mechanisms of these minerals [48]. Meanwhile, serum calcium and phosphorus concentrations were remarkably increased after LSSE treatment, due to its excellent supply of phosphorus and calcium and the ability of linolenic acid to reduce calcium elimination [49, 50].

The alternation of the antioxidant system in the PCOS group signifies the occurrence of cellular damage within the tissues [51]. This happens as a reaction to hyperglycemia causing a proinflammatory condition, which is linked to hyperandrogenism and insulin resistance causing the elevated formation of ROS, as demonstrated by malondialdehyde (MDA) and nitric oxide elevated concentrations and glutathione (GSH) reduced levels [52].

In this experiment, LSSE reduced MDA and NO and elevated GSH and CAT. This change was owed to the bioactive compounds such as phenolic compounds, phytosterols, tocols, and tocopherols in LSSE, enhancing the antioxidant system [15].

The elevated concentration of DNA fragmentation in tissues detected in the PCOS model suggests dysregulation of apoptotic pathways, potentially due to altered expression or activity of apoptosis-regulating factors [53]. The DNA fragmentation increased due to hormonal imbalances and oxidative stress associated with the condition, potentially contributing to ovarian dysfunction in PCOS [54]. Furthermore, obesity-related DNA damage negatively correlates with SHBG [55].

On the contrary, treatment with LSSE (500 mg/kg) showed excellent results in opposing DNA fragmentation compared to the untreated PCOS group, which could be owed to glucosinolates, which were proven to interact with the proteins associated with the DNA repair mechanisms [56].

The ovarian tissue histological examination validated the biochemical results, revealing significant structural alterations in the PCOS group. Key observations included a marked decrease in developing follicles and the formation of many atretic and large thin-walled cystic follicles, likely due to reduced FSH, elevated LH/FSH ratio, and abnormal androgen concentration [57]. Also, TNF-α induces granulosa cell apoptosis, which was noticed in the thin wall of the Graafian follicle [58]. Also, the low calcium contributed to the impairment of oocyte maturation by disrupting mitochondrial function and ATP production [59]. Dilated and congested blood vessels in the ovarian medulla were attributed to the high estrogen and NO concentrations [60, 61].

The histopathological findings in the uterine tissue sections of PCOS rats revealed significant alterations in the endometrial architecture. Notably, a marked reduction in endometrial glands was observed throughout the endometrial tissue, which may contribute to impaired endometrial receptivity and higher implantation failure rates [62]. The hyperandrogenic state induces oxidative stress and inflammation negatively impacting blood flow, which impairs uterine receptivity and compromises its ability to sustain a healthy pregnancy [63].

Conversely, the LSSE-treated groups exhibited a general improvement in ovarian and uterine tissue histoarchitecture, characterized by an increase in antral follicles and corpora lutea and a significant decrease in the atretic and cystic follicles. Also, the Graafian follicle has the same architecture and granulosa thickness as the control indicating better quality of oocytes, which could be attributed to the restoration of calcium aiding in normal oocyte maturation [64]. The blood vessels in the ovary showed a lower degree of dilation and congestion in a dose-dependent manner. LSSE-treated groups showed a slight enhancement in uterine histology, characterized by a thicker uterine wall and a higher number of endometrial glands than the PCOS group. All these protective effects can be attributed to the balanced hormonal concentrations, lower nitric oxide concentration induced by the anti-inflammatory, anti-apoptotic, antioxidant, and DNA-repairing properties of LSSE, due to its content of ferulic acid, gallic acid, coumaric acid, and caffeic acid [15].

Immunohistochemical analysis revealed markedly elevated expression of tumor necrosis factor alpha (TNF-α) in the PCOS group. This rise in TNF- α indicates an inflammatory response due to the innate immunity reaction to the damaged cells and apoptosis [65]. The concurrent upregulation of TNF- α underscores the profound impact of chronic low-grade inflammation on the reproductive tract in PCOS [66].

On the other hand, the TNF-α recorded a significant reduction in the LSSE-treated groups, which could be justified by its protective effect against inflammation, hyperglycemia, and hyperandrogenemia. Also, LSSE is a strong antioxidant, which promotes the anti-inflammatory effect by inhibiting the production of nuclear factor kappa-light-chain-enhancer of activated B cells (NF-κB) [67].

The current results showed that PCOS rats had lower mating and fertility indices, where PCOS is known to be accountable for decreased fertility [68]. These indices are affected by the hyperandrogenic state, imbalanced hormones, cystic follicles, and oxidative stress [69]. These alternations were reduced in the groups treated with LSSE in a dose-dependent manner. Additionally, nonsignificant changes were noticed in the maternal body weight across all groups, indicating no maternal toxicity, except for metformin, which showed the highest maternal body weight and could be due to the effect of metformin on renal filtration and net tubular transport during pregnancy, causing fluid retention [70].

The study findings showed no significant differences between the control, PCOS, and treated groups on the average number of corpora lutea, implantation sites, the weights of placenta and gravid uterus. On the other hand, the untreated PCOS rats had the highest preimplantation loss in all groups; this can be attributed to the low quality of oocytes and maturation issues or the thinning of the endometrium, leading to decreased receptivity. Elevated nitric oxide and TNF- α can disrupt the processes of endometrial decidualization and implantation, compromising endometrial receptivity [71]. Also, obesity with PCOS remains a risk factor for decreased rates of embryo implantation and live births and an increased risk of early abortion [72].

The insignificant increase in the corpora lutea number in the untreated PCOS group after the mating trials, although it had scored a significant decrease in the histological count, can be attributed to the mating process that was found to enhance the expression of hormonal and trophic factors, that can cause a slight improvement in the ovarian function [73].

Moreover, PCOS progeny outcome showed a significant increase in fetal mortality and morphological aberrations such as hematoma, which might be attributed to the elevated androgens and insulin that affect the intrauterine environment, potentially leading to complications during pregnancy [74]. Additionally, androgen excess during pregnancy can decrease rat fertility, where excess androgen at the early stage of pregnancy causes high reproductive toxicity, leading to the abnormality of ovarian morphology and functions in female offspring [75]. Oxidative stress can compromise the transfer of nutrients and oxygen to the fetus by the placenta, increasing the risk of hematoma and fetal death [76]. Fetal mortality can be attributed to increased oxidative ROS caused by hyperandrogenism, and insulin resistance affects placental formation, increases glycogen accumulation, and reduces angiogenesis [77].

The groups treated with LSSE showed no difference in the fetus's weight. No mortality or hematoma was recorded in either group, which indicates normal blood flow and placenta due to the balanced hormones. LSSE groups showed a higher number of fetuses than the PCOS group, with lower pre and postimplantation losses. That could be owed to the improved endometrial receptivity, allowing more embryos to be implanted successfully.

LSSE outperformed metformin in numerous parameters, including physical and serum biochemical parameters, while the antioxidant system and DNA damage showed similar results. LSSE-treated groups had fewer cystic follicles and decreased blood vessel congestion. Both therapies had little effect on uterine histoarchitecture. LSSE (500 mg/kg) and metformin had similar mating and fertility indices as LSSE (250 mg/kg). Still, the low dose had improved pregnancy outcomes with reduced preimplantation, no postimplantation loss, and more viable fetuses than the metformin group. However, metformin therapy caused increased maternal body weight for pregnant rats and fetal hematoma, which wasn't noticed in the LSSE-treated groups.

**Fig. 10:**
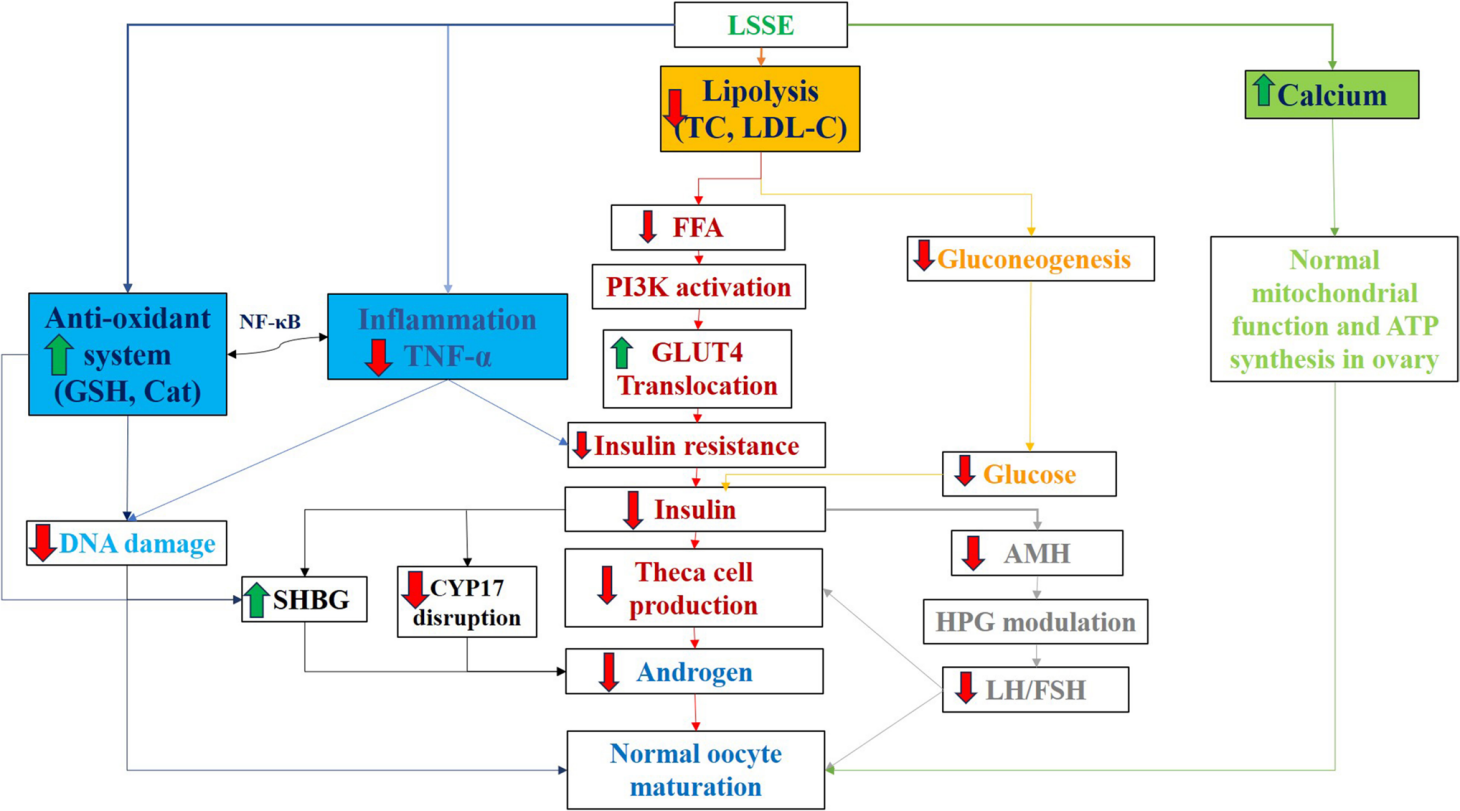
The protective mechanisms of LSSE against PCOS. LSSE: *Lepidium sativum* seed extract.TC: Total cholesterol, LDL: low-density lipoprotein, FFA: free fatty acids, PI3K: phosphatidylinositol-3 kinase, GLUT4: Glucose transporter type 4, GSH: glutathione, CAT: Catalase, TNF- α: tumor necrosis factor-alpha, NF-κB: Nuclear factor kappa-light-chain-enhancer of activated B cells, SHBG: sex hormone binding globulin, CYP17: Cytochrome P450 17alpha-hydroxylase/17,20-lyase, AMH: Anti-müllerian hormone, HPG: hypothalamic-pituitary–gonadal axis, FSH: follicle stimulating hormone, LH: luteinizing hormone

Figure 10 explains the protective mechanism of LSSE against PCOS with four different pathways, including inhibition of inflammation and lipolysis, enhancement of the antioxidant system, and calcium production. LSSE exhibited excellent antioxidant and anti-inflammatory activities. In addition, the inhibition of TNF-alpha stimulates the production of antioxidants through the NF-κB pathway. This change led to the stimulation of SHBG release and the inhibition of DNA damage caused in tissues. Another pathway is the inhibition of lipolysis (reduction of total cholesterol and LDL concentrations), causing a suppression of gluconeogenesis and glucose concentration, causing a decline in insulin.

Meanwhile, lipolysis reduction inhibits the FFA levels that activate PI3K, which stimulates the GLUT4 translocation, causing a decrease in insulin resistance, which reduces insulin production. Reduced insulin increases SHBG synthesis and inhibits CYP17, theca cells, androgen, and AMH. In addition, inhibiting AMH suppresses GnRH from the hypothalamus, which modulates pituitary gland sex hormone production, increasing FSH and decreasing LH, restoring hormonal balance in the ovary, reducing androgenic effect and allowing follicles to mature normally. Finally, the high mineral content of LSSE with calcium regulates ovarian mitochondrial ATP generation to normalize oocyte maturation.

## Conclusion

This study suggests that LSSE may protect against PCOS by improving physical parameters, hormonal imbalances, metabolic dysfunctions, antioxidant system, mating and fertility indices, pregnancy outcomes, and fetal morphology. In most measurements, LSSE's dose-dependent effects are best at (500 mg/kg), while the pregnancy outcomes were enhanced with the (250 mg/kg) dose. Metformin was less effective than LSSE in most parameters. In conclusion, LSSE's potential as a natural, multi-target PCOS treatment makes it an intriguing supplement to traditional PCOS treatments.

## Authors Contribution

M.A. and M.H. carried out the experiment and made significant contributions to the research concept, manuscript writing, statistical studies, and data interpretation. A.S. made substantial contributions to the experiment design, protocol, data interpretation, and adjusting the draft for important intellectual content. A.I. contributed to the study concept, design, data interpretation, and revising the paper for important intellectual content. All authors had full access to all study data and had final responsibility in the decision to submit for publication. The authors declare that all data were generated in-house and that no paper mill was used.

## Data Availability

The data supporting this study's findings are available from the corresponding author upon request.
